# A chromosome-level genome assembly and evolutionary analysis of *Coregonus ussuriensis* Berg

**DOI:** 10.1038/s41597-024-03642-0

**Published:** 2024-07-18

**Authors:** Tianqing Huang, Enhui Liu, Baorui Cao, Wenwen Li, Gaochao Wang, Wei Gu, Haibing Ma, Fulin Dong, Bingqian Wang, Gefeng Xu

**Affiliations:** 1 Key Laboratory of Freshwater Aquatic Biotechnology and Breeding, Ministry of Agriculture and Rural Affairs, Heilongjiang River Fisheries Research Institute, Chinese Academy of Fishery Sciences, Harbin, PR China; 2 Yantai Jinghai Marine Fishery Co Ltd, Yantai, PR China

**Keywords:** Genomics, Evolutionary genetics

## Abstract

*Coregonus ussuriensis* Berg, distributed widely in cold waters above 45° N latitude, is a savored freshwater whitefish that has been included in the list of endangered animals as a consequence of overfishing. Lack of genomic information seriously hampers evolutionary and genetic research on *C. ussuriensis* warranting the need to assemble a high-quality reference genome to promote its genetic breeding. We assembled and constructed a reference chromosome-level *C. ussuriensis* genome (sequence length, 2.51 Gb; contig N50 length, 4.27 Mb) using PacBio sequencing and Hi-C assembly technology, 3,109 contigs were assembled into scaffolds, resulting in a genome assembly with 40 chromosomes and a scaffold N50 length of 62.20 Mb. In addition, 43,320 protein-coding genes were annotated. The peak Ks position in the species comparison reflects the whole-genome replication event of *C. ussuriensis*. This chromosome-level genome provides reference data for further studies on the molecular breeding of *C. ussuriensis*.

## Background & Summary

**Fig. 1:**
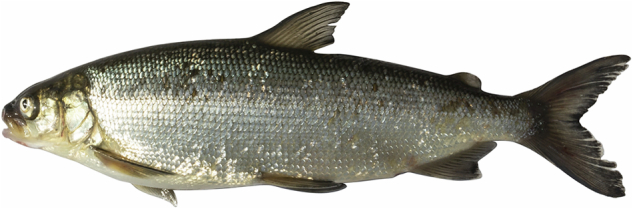
Picture of two-years-old female *Coregonus ussuriensis* Berg.

*Coregonus ussuriensis* Berg belong to the order Salmoniformes, family Salmonidae, subfamily Coregoninae, and genus *Coregonus*. They inhabit cold-water basins above 45° N latitude, such as Siberia and Sakhalin in Russia and Heilongjiang in China, which have typical migration characteristics. The body of *C. ussuriensis* is long, flat, and fusiform, with a shorter caudal stalk, shorter head, and a larger mouth in the terminal position. The eyes are larger and closer to the rostral end. The fish scale is round, large and easy to fall off. The back of the body is bluish-grey, and the side of the body is silver-white (Fig. 1)^1,2^. As one of the rare fishes, the meat of *C. ussuriensis* is delicate, has high nutritional and economic value, and is popular among consumers^3^. However, owing to the deterioration of the living environment, overfishing, and other factors, the *C. ussuriensis* resources have shown a significant decline and are included in the Red Book of Endangered Animals (Fish) of China^4^. For sustainability of *C. ussuriensis* genetic resources, research on its breeding and reproduction has been gradually undertaken.

Whole-genome sequencing of specific species is essential for solving practical problems in biological research and aquaculture. Genome sequencing technology is fast maturing with developments in science and technology^5^. The ray-finned fish have strong reproductive ability and adaptability; they constitute the most varied, abundant, and widely distributed vertebrate species^6^. Fish have gradually become ideal models for vertebrate genome analysis and gene function identification. More than 200 aquatic animal genomes have been sequenced and are available in the public database of the National Center for Biotechnology Information (NCBI)^7^. Accurate analysis of the fish genome can reveal essential regulatory genes for the desired economic traits, providing vital data for improving production and breeding efficiency^8,9^.

Recent research on *C. ussuriensis* has mainly focused on muscle nutritional quality, gonadal and embryonic development, reproductive performance, and pathogen infection^10–16^. However, reports on the genome of *C. ussuriensis* have been lacking, which seriously hampers research on genetic selection at the molecular level. To overcome this gap in knowledge, in this study, we assembled and constructed a reference chromosome-level genome of *C. ussuriensis* using PacBio sequencing and Hi-C assembly technology. The genome assembly had a total length of 2.51 Gb, with a contig N50 of 4.27 Mb and a scaffold N50 of 62.20 Mb. This reference genome provides essential data for developing molecular markers for economic traits and should help conserve and utilise the germplasm resources of *C. ussuriensis*.

## Methods

### Ethics statement

All experiments were approved by the Animal Husbandry Department of the Heilongjiang Animal Care and Use Committee. All fish involved in this research were bred following the guidelines of the Animal Husbandry Department of Heilongjiang, China.

### Sample collection and DNA extraction

Samples of *C. ussuriensis* were collected from Bohai Cold Water Fish Experimental Station of Heilongjiang Fisheries Research Institute (129° 04′ 64.7753′′ E; 44° 14′ 5.983′′ N). The liver tissue of the *C. ussuriensis* shown in Fig. 1 was collected and stored in liquid nitrogen for DNA extraction, genome library construction, and high-throughput sequencing. The CTAB-based extraction method was used to extract DNA from the liver tissue^17,18^. The DNA concentration was 254.0 ng/μL, and the OD260/280 value was 1.83. The quality and quantity of the extracted DNA were examined using a NanoDrop 2000 spectrophotometer (NanoDrop Technologies, Wilmington, DE, USA), Qubit dsDNA HS Assay Kit on a Qubit 3.0 Fluorometer (Life Technologies, Carlsbad, CA, USA) and electrophoresis on a 0.8% agarose gel, respectively.

### SMRTbell library construction and PacBio sequencing

The SMRTbell library was constructed using the SMRTbell Express Template Prep kit 2.0 (Pacific Biosciences). Briefly, 5 μg of the genomic DNA mentioned above was carried into the first enzymatic reaction to remove single-stranded overhangs, followed by treatment with repair enzymes to repair any damage that may be present on the DNA backbone. After DNA damage repair, the ends of the double-stranded fragments were polished and subsequently tailed with an A-overhang. Ligation with T-overhang SMRTbell adapters was performed at 20 °C for 60 minutes. Following ligation, the SMRTbell library was purified with 1X AMPure PB beads. The size distribution and concentration of the library were assessed using the FEMTO Pulse automated pulsed-field capillary electrophoresis instrument (Agilent Technologies, Wilmington, DE) and the Qubit 3.0 Fluorometer (Life Technologies, Carlsbad, CA, USA). Following library characterisation, 3 μg was subjected to a size selection step using the BluePippin system (Sage Science, Beverly, MA) to remove SMRTbells ≤25 kb. After size selection, the library was purified with 1 X AMPure PB beads. The FEMTO Pulse and the Qubit dsDNA HS reagents Assay kit assessed library size and quantity. Sequencing primer and Sequel II DNA Polymerase were annealed and bound to the final SMRTbell library, respectively. The library was loaded at an on-plate concentration of 35 pM using diffusion loading. SMRT sequencing was performed using a single 8 M SMRT Cell on the Sequel II System with Sequel II Sequencing Kit^19,20^.

### Hi-C library construction and sequencing

Four steps were performed for the *In situ* Hi-C library construction. The first was formaldehyde cross-linking; 1 g of the same *C. ussuriensis* shown in Fig. 1 was cross-linked for 10 min with 1% fresh formaldehyde and quenched with 0.2 M final concentration glycine for 5 min. The second was the cell lysis; the cross-linked cells were subsequently lysed in lysis buffer (10 mM Tris-HCl (pH 8.0), 10 mM NaCl, 0.2% NP40, and complete protease inhibitors (Roche)). The extracted nuclei were re-suspended with 150 μl 0.1% SDS and incubated at 65 °C for 10 min, then SDS molecules were quenched by adding 120 μl water and 30 μl 10% Triton X-100, and incubated at 37 °C for 15 min. The DNA in the nuclei was digested by adding 30 μl 10x NEB buffer 2.1 (50 mM NaCl, 10 mM Tris-HCl, 10 mM MgCl_2_, 100 μg/ml BSA, pH 7.9) and 150U of MboI, and incubated at 37 °C overnight. The third step was the digestion and biotin labelling. After the MboI enzyme was inactivated at 65 °C for 20 min, the cohesive ends were filled in by adding 1 μl of 10 mM dTTP, 1 μl of 10 mM dATP, 1 μl of 10 mM dGTP, 2 μl of 5 mM biotin-14-dCTP, 14 μl water and 4 μl (40 U) Klenow, and incubated at 37 °C for 2 h. The fourth step was the ligation and DNA purification, 663 μl water, 120 μl 10 x blunt-end ligation buffer (300 mM Tris-HCl, 100 mM MgCl2, 100 mM DTT, 1 mM ATP, pH 7.8), 100 μl 10% Triton X-100 and 20 U T4 DNA ligase were added to start proximity ligation. The ligation reaction was placed at 16 °C for 4 h. After ligation, the cross-linking was reversed by 200 μg/mL proteinase K (Thermo) at 65 °C overnight. According to manufacturers’ instructions, DNA purification was achieved through the QIAamp DNA Mini Kit (Qiagen). Sequencing was performed after the library quality was verified using a BGI MGISEQ-2000 platform (PE150) sequencer.

### RNA extraction and transcriptome sequencing

In total, five fish were taken for RNA extraction and transcriptome sequencing; the heart, liver, spleen, intestine, kidney, and muscle tissues of each fish were mixed to extract RNA for sequencing. Total RNA was extracted using the Trizol (Invitrogen, CA, USA), RNA purity and integrity was monitored by NanoDrop 2000 spectrophotometer (NanoDrop Technologies, Wilmington, DE, USA) and a Bioanalyzer 2100 system (Agilent Technologies, CA, USA). RNA contamination was assessed by 1.5% agarose gel. RNA concentrations ranged from 587.0 to 2475.8 ng/μL, and the OD260/280 values ranged from 1.98 to 2.06. The integrity of RNA detection showed that the RNA integrity number ranged from 8.7 to 10, and 28 S/18 S values ranged from 0.9 to 1.8. Transcriptome sequencing of qualified RNA was performed on an Illumina NovaSeq. 6000 platform and the results were used for gene prediction.

### Genome survey analysis

Before genome assembly, SOAPnuke v2.1.0^21^ was used to control the quality of the DNA sequencing library. Reads containing joints and low-quality reads were removed to obtain 196.84 Gb of clean reads. Based on the effective sequence information, the K-mer analysis was performed using the GCE v1.0.2 software^22^ to estimate the genome size, heterozygosity rate, repeat sequences, and other information. The K value was set at 17 to ensure sufficient generation of K-mer species to cover the entire genome. The results of K-mer analysis showed that the estimated genome size was 2560.3 Mb, heterozygosity rate was 0.66%, proportion of repeated sequences was 73.32%, and GC content was approximately 42.75%.

### Genome assembly

**Fig. 2:**
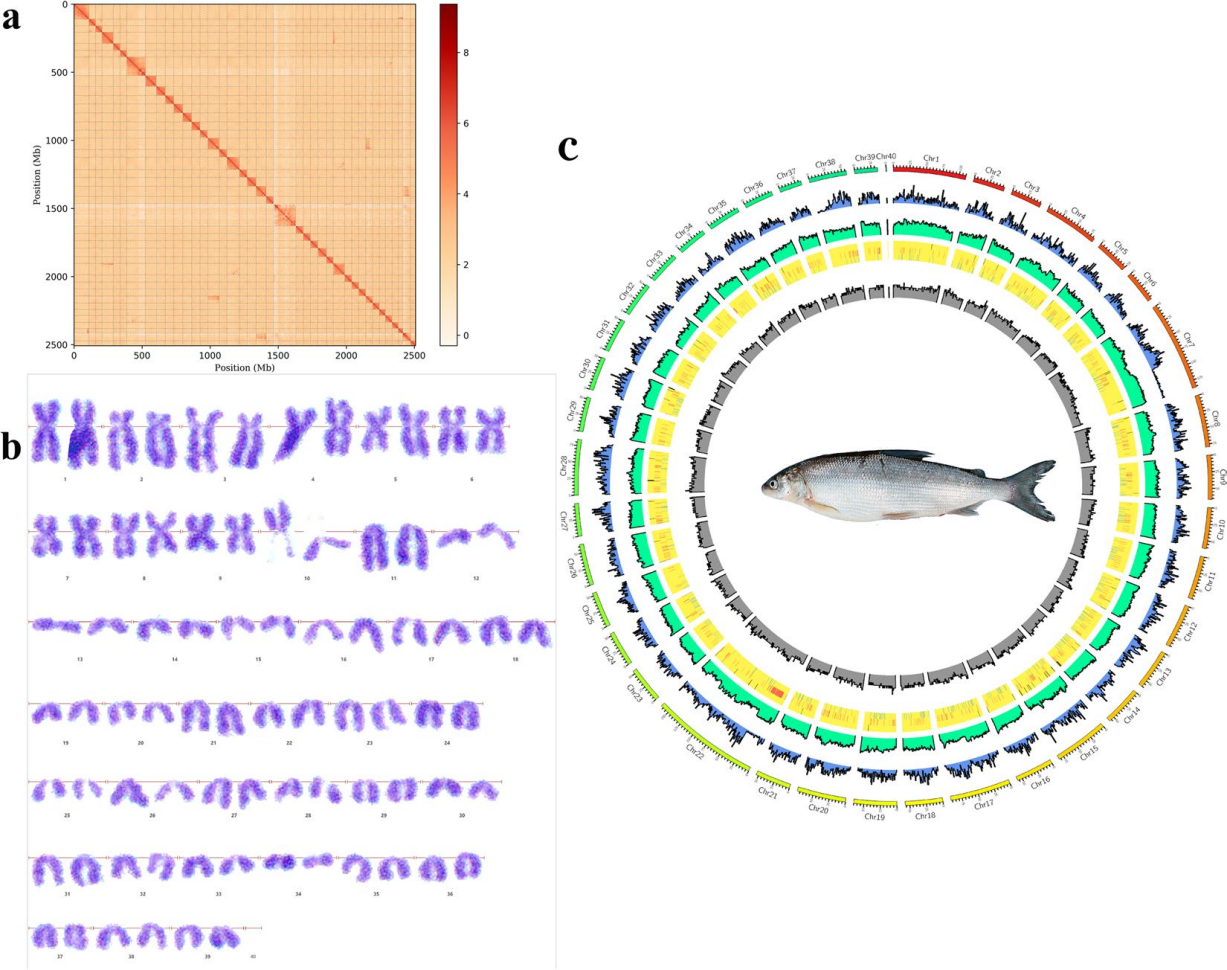
Characteristics of the *Coregonus ussuriensis* genome. (**a**) Hi-C intrachromosomal contact map of the *C. ussuriensis* genome assembly; the frequency of interactions was calculated using a window size of 500 kb. The color bar shows the contact density from low (white) to high (red). (**b**) Cytological karyotype map of *C. ussuriensis*. (**c**) Circos plot of the *C. ussuriensis* genome assembly. The tracks from outside to inside are 40 chromosome-level scaffolds; gene density; repeat density; region of ncRNAs (the first little ring is rRNA distribution, the second small ring is snRNA distribution, the third small ring is miRNA distribution); and GC content.

The Trimmomatic software was used to trim the original data to reduce the number of adapter sequences and low-quality fragments^23^. Long-read data of 282.97 Gb (clean reads) were assembled using the assembly software, NextDenovo^24^. The genome sequence was assembled after error correction and removal of redundancy. Finally, the 3D-DNA software was used to cluster and construct an interaction matrix. The Juicebox software^25^ was used to build the chromosome interaction map, and JuiceBox was used for visual error correction. The assembled genome was 2,627.19 Mb in size, and contained 3,109 contigs and 4.27 Mb contig N50 (Table 1). Hi-C data analysis was performed to assemble further the contigs obtained from the initial assembly to the scaffold level. Finally, 2.51 Gb of the genome sequence was obtained, the scaffold N50 was 62.20 Mb, and 95.45% of the original assembly sequence was attached to 40 pairs of chromosomes (Fig. 2a), it was noticed that there was the smallest scaffolds in chr40, only 1.12 Mb in size, which was consistent with the small super-scaffold of *Coregonus* sp. *Balchen*^26^. The microchromosomes were difficult to find histologically, so the chromosomal karyotype were 39 pairs (Fig. 2b). Specific information regarding the chromosomes is shown in Table 2. A chromosomal circle diagram was drawn based on the 40 constructed chromosomes (Fig. 2c) using the CIRCOS^27^. Our input data comprised annotated gene and ncRNA gff files, alongside the genome sequence and its masked version post-repetition sequence filtration. Circos generated a comprehensive circular plot, delineated into five concentric layers. The outermost layer represents the chromosomes, followed inwardly by gene density, repeat sequence density, and ncRNA regions—subdivided into rRNA, snRNA, and miRNA zones for detailed visualization. Due to the excessive quantity of tRNA annotations, their representation was omitted in this iteration as we are currently refining the tRNA annotation results. The innermost layer illustrates the GC content.

**Table 1:** Summary of genome sequencing, assembly, and annotation results.

**PacBio sequencing**	
Contig N50 (Mb)	4.27
Contig number	3109
Contig total length (Mb)	2627.19
**Hi-C data**	
Scaffold N50 (Mb)	62.20
Scaffold number	494
Number of Superscaffold (chromosome)	40
Total length of Superscaffold (Gb)	2.51
Integration efficiency of Hi-C map (%)	95.45
**Scaffolded assembly BUSCOs**	
Complete(%)	95.00
Complete and single-copy (%)	50.50
Complete and duplicated (%)	44.50
Fragmented(%)	1.20
Missing(%)	3.80

**Table 2:** Summary information for each chromosome of *Coregonus ussuriensis*.

Superscaffold	Number of Contigs	Length of Contigs	Length of Superscaffold
Superscaffold 1	118	110,382,044	110,440,544
Superscaffold 2	52	48,041,282	48,066,782
Superscaffold 3	51	47,332,622	47,357,622
Superscaffold 4	43	83,137,478	83,158,478
Superscaffold 5	53	50,257,611	50,283,611
Superscaffold 6	60	48,903,990	48,933,490
Superscaffold 7	217	136,521,738	136,629,738
Superscaffold 8	100	79,431,272	79,480,772
Superscaffold 9	37	66,834,325	66,852,325
Superscaffold 10	46	62,172,604	62,195,104
Superscaffold 11	32	65,528,461	65,597,961
Superscaffold 12	50	66,188,646	66,213,146
Superscaffold 13	39	60,486,759	60,505,759
Superscaffold 14	61	58,145,255	58,175,255
Superscaffold 15	51	82,558,298	82,583,298
Superscaffold 16	46	57,494,056	57,516,556
Superscaffold 17	84	91,857,605	91,899,105
Superscaffold 18	44	56,759,606	56,817,106
Superscaffold 19	43	65,128,621	65,149,621
Superscaffold 20	39	74,228,189	74,247,189
Superscaffold 21	30	53,849,652	53,864,152
Superscaffold 22	423	163,935,738	164,146,738
Superscaffold 23	39	54,295,076	54,314,078
Superscaffold 24	34	52,952,213	52,968,713
Superscaffold 25	29	54,218,384	54,232,384
Superscaffold 26	68	63,016,922	63,050,422
Superscaffold 27	38	49,552,441	49,570,941
Superscaffold 28	130	84,176,193	84,240,693
Superscaffold 29	45	52,298,530	52,320,530
Superscaffold 30	47	51,135,089	51,158,089
Superscaffold 31	34	51,195,761	51,212,261
Superscaffold 32	107	51,790,456	51,843,456
Superscaffold 33	39	47,654,661	47,673,661
Superscaffold 34	36	44,370,636	44,388,136
Superscaffold 35	43	49,725,274	49,746,274
Superscaffold 36	37	44,638,141	44,656,141
Superscaffold 37	13	34,249,620	34,255,620
Superscaffold 38	150	57,034,453	57,108,953
Superscaffold 39	46	34,865,653	34,888,153
Superscaffold 40	1	1,120,304	1,120,304
Total	2,655	2,507,555,659	2,508,863,159

### Repeat sequence annotation

The repeat sequences of the genome were predicted using homologous prediction based on the RepBase library (http://www.girinst.org/repbase) in conjunction with de novo prediction. RepeatMasker (open-4.09)^28^ and RepeatProteinMask (open-4.09) were used to search for tandem repeats in the RepBase (release 21.01)^29^. RepeatModeler (v open 1.0.11)^30^ and LCR-Finder (v 1.0.5) software^31^ were used to create the de novo (de novo sequencing) repeat sequence database. Finally, we used the RepeatMasker (open 4.0.9) and TRF software to annotate the repeat sequences using TE and de novo libraries. After removing the overlapping non-redundant parts, the combined annotation results were used for statistical analysis. We also identified the length of DNA transposons was 672.59 Mb, the long interspersed repeated sequences (LINE) was 353.29 Mb, the short interspersed repeated sequences (SINE) was 17.57 Mb, the long terminal repeat (LTR) was 177.46 Mb. All transposable element (TE) sequences accounted for 58.63% of the whole genome sequence (Table 3). The repeat sequence density statistics are shown in Fig. 2c.

**Table 3:** Classification statistics of repeated sequences in the genome of *Coregonus ussuriensis*.

	RepeatMasker TEs		RepeatProteinMask TEs		*de novo*		Combined TEs	
	Length (bp)	In Genome %	Length (bp)	In Genome %	Length (bp)	In Genome %	Length (bp)	In Genome %
DNA	484,865,736	18.45	14,476,170	0.55	561,899,847	21.38	672,589,088	25.59
LINE	188,796,186	7.18	231,588,715	8.81	288,043,035	10.96	353,289,331	13.44
SINE	7,895,642	0.30	0	0.00	10,977,647	0.42	17,565,547	0.67
LTR	105,163,680	4.00	93,023,849	3.54	124,362,248	4.73	177,460,740	6.75
Unknown	4,477,644	0.17	0	0.00	378,073,721	14.38	378,551,655	14.40
Total TE	764,728,462	29.09	339,010,869	12.90	1,346,033,800	51.21	1,541,137,731	58.63

### Gene function prediction and annotation

**Fig. 3:**
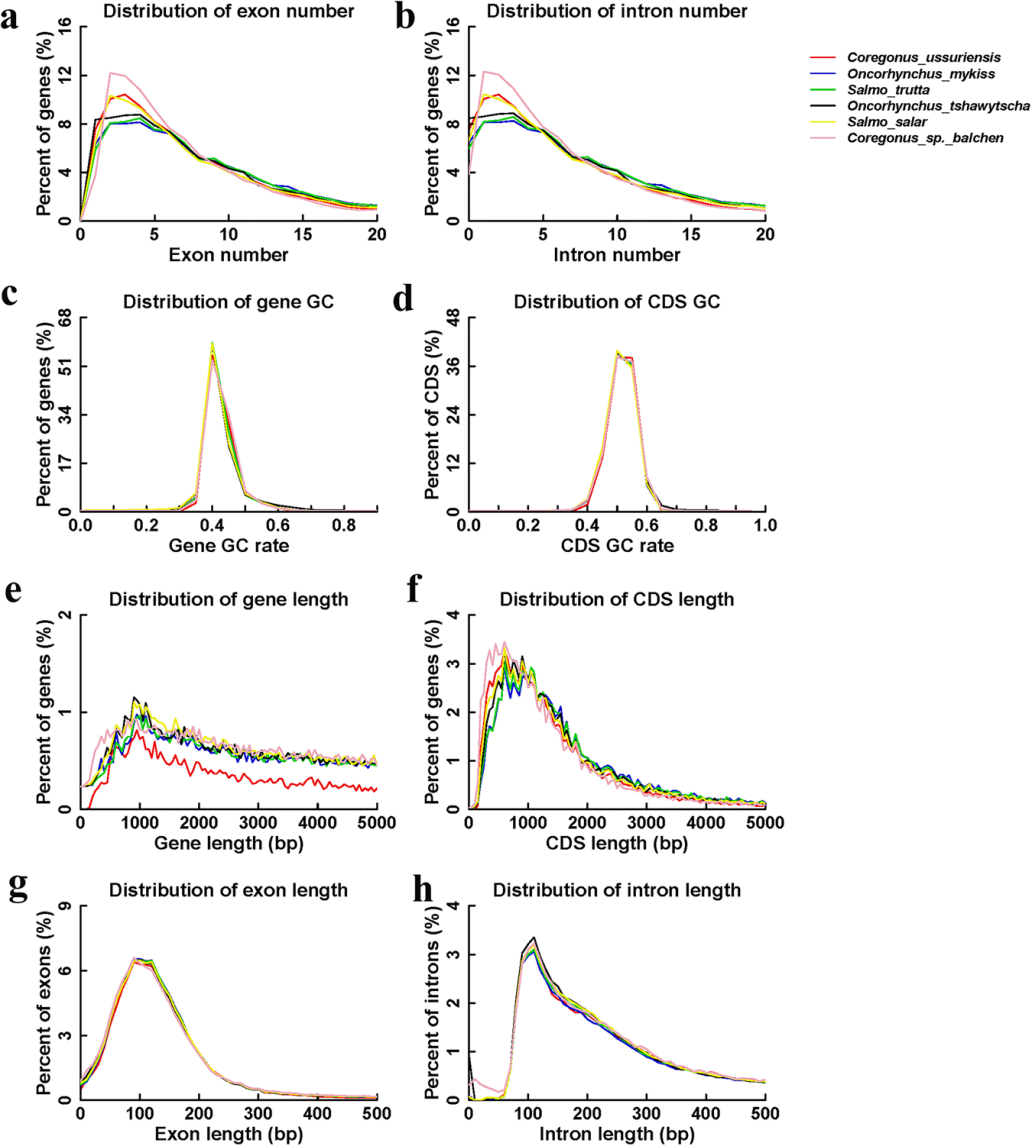
Statistical map of gene sets for gene structure prediction. (**a**) Exon number. (**b**) Exon length. (**c**) Intron number. (**d**) Intron length. (**e**) Gene length. (**f**) GC-content of genes. (**g**) Coding sequence (CDS) length. (**h**) GC-content of CDS.

We combined the de novo prediction, homologous annotation, and RNA-Seq methods to predict and annotate protein-coding genes in the genome of *C. ussuriensis*. The Augustus (v3.3), GlimmerHMM (v3.0.4), and Genscan software were used for ab initio gene prediction. For homologous annotation, we selected five closely related species, *Oncorhynchus mykiss* (GCF_013265735.2)^32^, *Salmo trutta* (GCF_901001165.1)^33^, *Oncorhynchus tshawytscha* (GCF_002872995-1)^34^, *Salmo salar* (GCF_000233375-1)^35^, and *Coregonus* sp. *Balchen* (GCA_902810595-1)^26^ for comparison with the genome of *C. ussuriensis* using the TblastN software (with an e-value cutoff of 1e-5)^36^. The Exonerate software (v2.2.0; parameters: -model protein2 genome -percent 20-minintron 10, -maxintron 50000)^37^ was used for precise homologous genome sequence splicing of the matched proteins. For RNA-seq prediction and annotation, the Trinity^38^ software was used to assemble RNA-seq data from six tissues, namely the heart, liver, spleen, intestine, stomach, and muscle, and the PASA software was used to predict the gene structure. A total of 43,320 protein-coding genes were predicted and annotated, with an average length of 19,815.01 bp, average coding sequence length of 1,524.46 bp for each gene, and average exon number of 8.68 (Table 4 and Fig. 3). It is worth noting that *C. ussuriensis* had shorter gene lengths, shorter CDSs and less exons, but the number of genes was the largest, probably because of gene redundancy and even functional differentiation after duplication^39^. SwissProt, TrEMBL, KEGG, InterPro, GO, NR, and other protein databases were used to annotate protein functions of the gene prediction sets. Among them, 37,987 genes were annotated to the InterPro, 29,061 genes to the GO, 42,922 genes to the KEGG_ALL, 26,747 genes to the KEGG_KO, 39,392 genes to the Swissprot, 42,973 to TrEMBL, and 43,048 to the NR. A total of 43,066 protein-coding genes were annotated in the genome of *C. ussuriensis*, accounting for 99.41% of the predicted genes. The annotation results for each database are presented in Table 5.

**Table 4:** Statistical results of the predicted genes in the genome of *Coregonus ussuriensis*.

Gene set		Number	Average gene length (bp)	Average CDS length (bp)	Average exon per gene	Average exon length (bp)	Average intron length (bp)
De novo	AUGUSTUS	58,256	13,148.63	1,234.9	6.38	193.5	2,213.65
Homolog	O.mykiss	62,872	18,065.5	1,361.8	7.26	187.49	2,666.99
	S.trutta	63,494	17,546.53	1,348.19	7.2	187.24	2,612.43
	O.tshawytscha	60,398	16,259.77	1,272.63	6.96	182.92	2,515.83
	S.salar	66,990	15,685.94	1,270.17	6.72	188.92	2,518.71
	C.sp.	75,572	10,658.6	1,092.15	5.83	187.32	1,980.52
trans.orf/RNAseq		16,552	19,325.88	1,259.22	8.19	311.2	2,334.17
MAKER		44,617	17,830.37	1,489.94	8.36	208.09	2,187.03
PASA		43,320	19,815.01	1,524.46	8.68	233.31	2,317.49

**Table 5:** Statistical results of gene function annotation.

Type		Number	Percent(%)
Total		43,320	
Annotated	InterPro	37,987	87.69
	GO	29,061	67.08
	KEGG_ALL	42,922	99.08
	KEGG_KO	26,747	61.74
	Swissprot	39,392	90.93
	TrEMBL	42,973	99.2
	NR	43,048	99.37
Annotated		43,066	99.41
Unannotated		254	0.59

### Annotation of non-coding RNA

Noncoding RNAs include tRNAs, rRNAs, miRNAs, and snRNAs. tRNAs were annotated based on their structural characteristics with tRNAscan-SE (v1.3.1)^40^ using the default parameters. Owing to their high conservation, the rRNAs of related species are usually selected as the reference sequences. BLASTN (v2.6.0) was used to find the rRNA sequences in the genome. The covariance model in Rfam (v14.1) was used to predict miRNA and snRNA sequences in the genome using the INFERNAL (v1.0) software^41^. A total of 770 miRNAs, 150,183 tRNAs, 976 rRNAs, and 1,828 snRNAs were annotated in the *C. ussuriensis* genome (Table 6 and Fig. 2c).

**Table 6:** Statistical results of noncoding RNA annotation.

Type		Copy Number	Average length(bp)	Total length(bp)	% of genome
miRNA		770	85.1	65,529	0.0025
tRNA		150,183	75.61	11,354,996	0.432
rRNA	rRNA	976	123.46	120,501	0.0046
	18 S	13	984.46	12,798	0.0005
	28 S	0	0	0	0
	5.8 S	8	133	1,064	0
	5 S	955	111.66	106,639	0.0041
	8 S	0	0	0	0
snRNA	snRNA	1,828	126.66	231,543	0.0088
	CD-box	431	103.64	44,670	0.0017
	HACA-box	344	140.05	48,178	0.0018
	splicing	883	141.12	124,610	0.0047
	scaRNA	36	189.22	6,812	0.0003

### Genome collinearity analysis

**Fig. 4:**
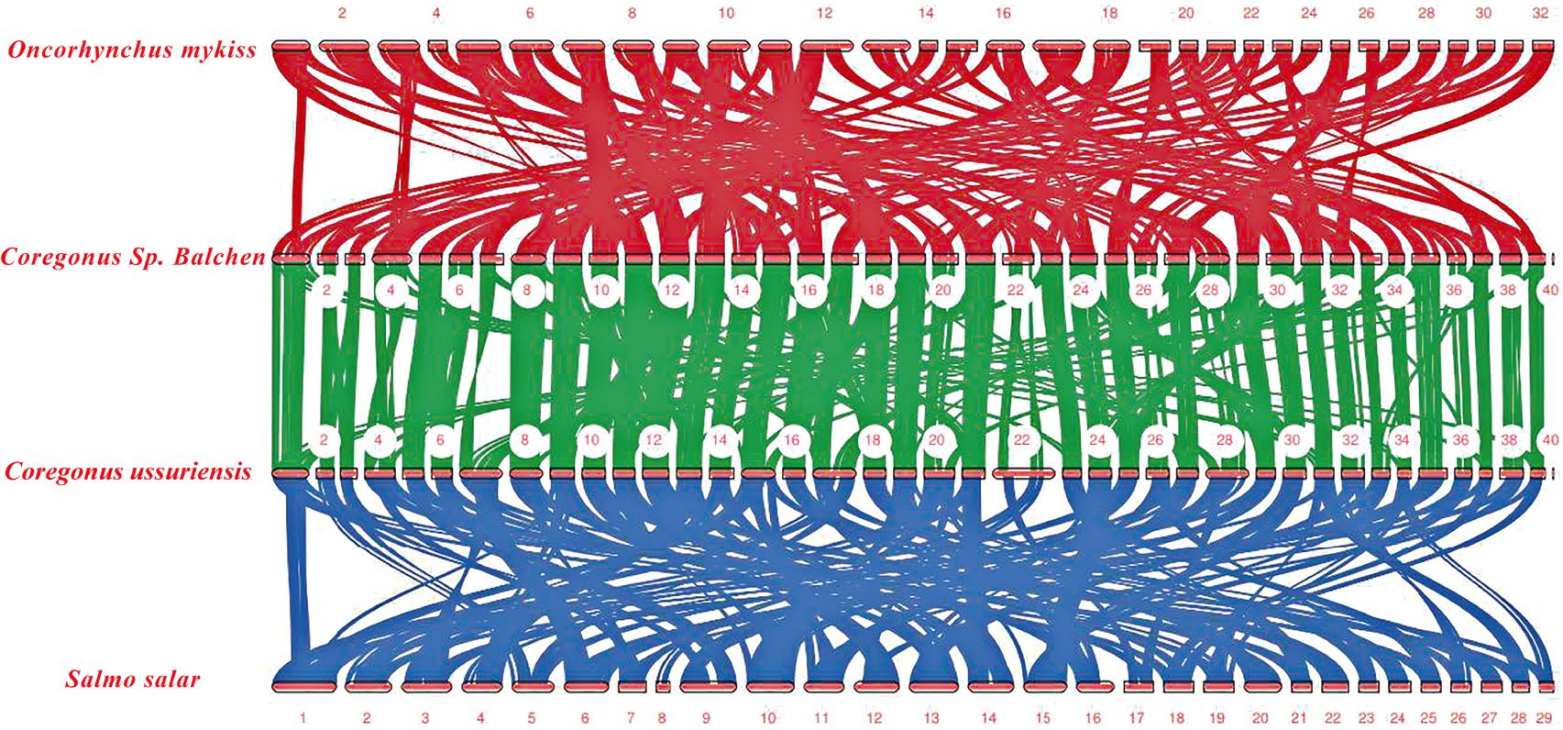
Collinearity analysis of reference genomes of *Coregonus ussuriensis* and other homologous species (*Oncorhynchus mykiss* vs. *Coregonus* sp. *Balchen* vs. *Coregonus ussuriensis* vs. *Salmo salar*).

The genome sequences of *O. mykiss*, *S. salar*, and *Coregonus* sp. *Balchen* were selected as references for comparison with the genome sequence of *C. ussuriensis*. The comparison and sequencing software used was Mummer (v4.0.0 beta2)^42^. The JCVI software^43^ was used to sequence the results and build a genomic collinear map (Fig. 4). The collinearity analysis revealed that *C. ussuriensis* and *Coregonus* sp. *Balchen* had the highest genomic homology with the other species. The super-scaffolds of *C. ussuriensis* were numbered to match with *C. sp Balchen*^26^ for consistency. The linear section indicates the large homologous fragments of the genome sequence within a species or between two species as a consequence of copy or species differentiation. The functions and sequences of genes in the homologous fragments are conserved. The MCScan software (http://chibba.agtec.uga.edu/duplication/mcscan/) was used to search for linear sections of the genome between these species. The plot figures of chromosome-by-chromosome comparison to *C.sp.Balchen*, *O. mykiss* and *S. salar* were shown as Figs S1-S3.

**Fig. 5:**
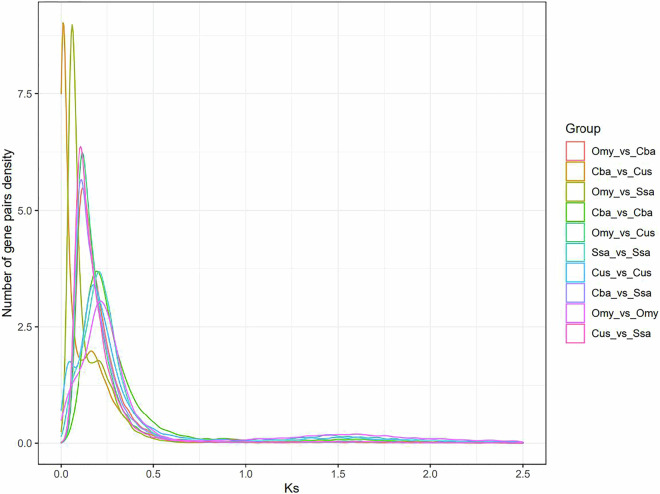
The Ks value (mutation rate of the homologous site) distribution diagram. *Cus: Coregonus ussuriensiss*; *Omy: Oncorhynchus mykiss*; *Cba: Coregonus* sp. *Balchen*; *Ssa: Salmo salar*.

The Ks value (mutation rate of the homologous site) of the gene pairs in the collinear segment was calculated. The Ks value can reflect the relative species differentiation and whole-genome replication events in the evolution of a species. The peak Ks position in the species comparison reflects the whole-genome replication event of the species^44^. According to the Ks value distribution map, the horizontal coordinate represents the Ks value and the vertical coordinate represents the number of gene pairs. By comparing the two peaks, it was possible to distinguish between the earlier and later stages of genome-wide replication and relative species differentiation (Fig. 5). As is evident from the figure, a whole genome replication (WGD) event occurred near Ks values of 0.1246, 0.2951, 0.1305, and 0.1246 for *C. ussuriensis*, *O. mykiss*, *S. salar*, and *Coregonus* sp. *Balchen*, respectively. Species differentiation occurred at a Ks value of 0.1650 for *Coregonus* sp. *Balchen* and *O. mykiss*, whereas it occurred at a Ks value of 0.1485 for *Coregonus* sp. *Balchen*. Species differentiation between *C. ussuriensis* and *S. salar* occurred at a Ks value of 0.1645. All Ks peaks were located between 0.12 and 0.3, demonstrating that they underwent whole-genome duplication, giving rise to tetraploid genomes with salmon-specific 4 R whole-genome duplications identical to those of *S. salar*, *O. mykiss* and *O. kisutch*^45^. The duplicated count in Busco (Table 1) and the off-target HiC hits (Fig. 2a) were also indicative of residually tetraploid regions, which providing further evidence that a burst of WGD occurred.

## Data Records

The BGI-SEQ, Pacbio, and Hi-C sequencing data used for genome assembly were deposited in the NCBI Sequence Read Archive database with accession numbers SRR25248458^46^, SRR25343505^47^, and SRR25494054^48^ under the BioProject accession number of PRJNA1000111.

The whole genome sequence project has been deposited at GenBank under the accession JAVHNI000000000^49^.

The transcriptomic sequencing data were stored under accession numbers SRR17306694-SRR17306711^50^.

The attachment results of BGI, Pacbio and Hi-C sequencing, gene annotation and evolutionary analysis were deposited in the Figshare^51^ database.

## Technical Validation

### Genome assembly assessment

To validate the accuracy and completeness of the genome assembly, Minimap2^28^ (v2.5, default parameters) was first used to compare the three-generation sequencing data with the assembled genome of *C. ussuriensis*. The comparison rate, extent of genome coverage, and depth distribution of reads were calculated, and 94.55% of the reads were mapped to contigs, with an average sequencing depth of 84.86× and an average genome coverage of 99.91%. Bases with sequencing depths greater than 10× accounted for 99.14% and those with sequencing depths greater than 20× accounted for 98.51% of the total number of bases. We assessed the integrity of the genome based on single-copy homologous genes from the OrthoDB set using the BUSCO software (v3.0.2) (https://gitlab.com/ezlab/busco), and the vertebrata_odb9 gene sets were chosen for BUSCO assessment. A total of 2,457 genes were compared using BUSCO (95.00%, complete), of which 1,306 were single-copy genes (50.50%, complete and single-copy), 1,151 were duplicated genes (44.50%, complete and duplicated), 31 were partially duplicated genes (1.20%, fragmented), and 98 were not duplicated (3.80%, missing). The genome assembly and annotation are summarised in Table 1. These results indicated that the genome assembly of *C. ussuriensis* was complete and of high quality.

### Karyotype analysis of C. ussuriensis

To verify the correctness of the C. ussuriensis genome assembly using Hi-C data, we fixed and stained the chromosomes and confirmed the chromosome number. For karyotyping, C. ussuriensis (n = 20) was raised at 18 °C for a week before sampling. The body weight of the experimental fish was 30 ± 0.8 g. Our preliminary experiments obtained the best chromosome fixation with phytohemagglutinin (PHA) and colchicine injected under the left pectoral fin. Sample preparation: Ten micrograms of PHA per gram of fish was injected; colchicine (3 mg/g of fish) was injected 24 h after the PHA injection. The samples were collected 4 h after colchicine injection.

The gill arches were cut and placed in water. The kidney tissue (whole kidney) was then collected immediately, washed two or three times with normal saline (85% NaCl solution), cut into pieces, and placed in a 10 mL beaker (approximately 8 mL of normal saline was added to the beaker). Cell low-osmosis: The filtrate obtained after filtration through 100 mesh gauze was placed in a 10 mL tube and centrifuged at 1200 rpm for 8 min. The supernatant was discarded, the pellet was gently dislodged, and the cells were incubated for 50 min in 6 mL of a hypotonic solution (0.075 mol/L KCl solution). Chromosome fixation: The above samples were mixed with 500 μL of a fixing liquid (methanol: glacial acetic acid = 3:1), centrifuged at 1200 rpm for 8 min, and the supernatant was discarded. After gently dislodging the pellet, 6 mL of the fixing solution was added, and the chromosome fixation was allowed to proceed for 20 minutes; this procedure was repeated three times. Drop slides and staining: The sample liquid (3¬–8 drops) was dropped onto a slide from a height and spread on the slide by gently blowing the liquid. The slide was passed over an alcohol lamp until the liquid almost dried, after which it was air dried with the side having the cells placed upright. Finally, the slide was placed with cells facing down on a staining plate for 30 min, rinsed with tap water, dried in air, and observed under a light microscope. By analysing with the Argus software, the microchromosomes were not visible under ordinary microscopes, so the karyotype analysis showed 39 pairs of chromosomes.

### Supplementary information

Tables

## Data Availability

All commands and pipelines used in data processing were executed according to the manuals and protocols of the corresponding bioinformatics software. No specific codes were developed for this study.

## References

[1] Liu E (2022). Molecular characterisation and antibacterial immunity functional analysis of the antimicrobial peptide hepcidin from Coregonus ussuriensis berg. Fish & Shellfish Immunology..

[2] Bochkarev NA (2017). The sympatric whitefishes Coregonus ussuriensis and C. chadary from the Amur River basin: Morphology, biology and genetic diversity. Fundam Appl Limnol..

[3] Wang J (2018). Evaluation of nutritive quality and nutrient components in the muscle of Coregonus ussuriensis berg. Journal of Guangdong Ocean University..

[4] Wang, S. China Red Data Book of Endangered Animals: Pisces (Science Press, 1998)

[5] Fritz A (2019). Chromosome territories and the global regulation of the genome. Genes, Chromosomes and Cancer..

[6] Ahmad S (2022). Fish genomics and its impact on fundamental and applied research of vertebrate biology. Reviews in Fish Biology and Fisheries..

[7] Lu G, Luo M (2020). Genomes of major fishes in world fisheries and aquaculture: Status, application and perspective. Aquaculture and Fisheries..

[8] Wang, J. *et al*. First Genomic Prediction of Single-Step Models in Large Yellow Croaker. Mar Biotechnol (NY). Jul 6, 10.1007/s10126-023-10229-0 (2023). 10.1007/s10126-023-10229-0 37410311

[9] Sinclair-Waters M (2022). Refining the genomic location of single nucleotide polymorphism variation affecting Atlantic salmon maturation timing at a key large-effect locus. Mol Ecol..

[10] Dong C (1997). The tentative studies on reproduction population structure of Coregonus ussurinsis. Chinese Journal of Fisheries..

[11] Ma B, Shi L, Dong C (2003). Biochemical genetic structure in Coregonus ussurinsis Berg. Journal of Fishery Sciences of China..

[12] Liang L, Chang Y, Dong C (2004). Analysis of genetic diversity for Coregonus ussurinsis Berg in Heilongjiang River. Journal of Fishery Sciences of China..

[13] Li P (2015). Fecundity of Coregonus ussurinsis in the Heilongjiang River, China. Journal of Fishery Sciences of China..

[14] Wang J (2018). Evaluation of Nutritive Quality and Nutrient Components in the Muscle of Coregonus ussuriensis. Journal of Guangdong Ocean University..

[15] Shi X (2020). Observation on Embryo Development of Whitefish Coregonus ussuriensis Berg in Heilongjiang River. Oceanologia et Limnologia Sinica..

[16] Liu E (2022). Cloning and tissue expression of liver-expressed antimicrobial peptide Leap-2 in Coregonus ussuriensis exposed to bacterial infection. Journal of Dalian Ocean University..

[17] Jones AS (1953). The isolation of bacterial nucleic acids using cetyltrimethylammonium bromide (cetavlon). Biochim Biophys Acta..

[18] Porebski S, Bailey LG, Baum BR (1997). Modification of a CTAB DNA extraction protocol for plants containing high polysaccharide and polyphenol components. Plant molecular biology reporter..

[19] Zhang T, Li M, Zhan Y, Fan G (2020). Dataset of full-length transcriptome assembly and annotation of apocynum venetum using pacbio sequel II. Data Brief..

[20] Kingan S (2019). A high-quality genome assembly from a single, field-collected spotted lanternfly (Lycorma delicatula) using the PacBio Sequel II system. Gigascience..

[21] Chen Y (2018). SOAPnuke: a MapReduce acceleration-supported software for integrated quality control and preprocessing of high-throughput sequencing data. Gigascience..

[22] Liu B (2013). Estimation of genomic characteristics by analysing k-mer frequency in de novo genome projects. Quantitative Biology..

[23] Bolger AM, Lohse M, Usadel B (2014). Trimmomatic: a flexible trimmer for Illumina sequence data. Bioinformatics..

[24] Hu, J. *et al*. NextDenovo: an efficient error correction and accurate assembly tool for noisy long reads. *Genome Biology*. **25**(1), (2024). 10.1186/s13059-024-03252-4 PMC11046930 38671502

[25] Durand N (2016). Juicer provides a one-click system for analysing loop-resolution Hi-C experiments. Cell systems..

[26] De-Kayne R, Zoller S, Feulner PGD (2020). A de novo chromosome-level genome assembly of Coregonus sp. “Balchen”: One representative of the Swiss Alpine whitefish radiation. Mol Ecol Resour..

[27] Krzywinski MI (2009). Circos: An information aesthetic for comparative genomics. Genome Research..

[28] Li H (2018). Minimap2: pairwise alignment for nucleotide sequences. Bioinformatics..

[29] Chen N (2004). Using Repeat Masker to identify repetitive elements in genomic sequences. Current Protocols in Bioinformatics..

[30] Flynn JM (2020). RepeatModeler2 for automated genomic discovery of transposable element families. Proceedings of the National Academy of Sciences..

[31] Xu Z, Wang H (2007). LTR_FINDER: an efficient tool for the prediction of full-length LTR retrotransposons. Nucleic acids research..

[32] Gao G (2021). A long reads-based de-novo assembly of the genome of the Arlee homozygous line reveals chromosomal rearrangements in rainbow trout. G3 (Bethesda)..

[33] Hansen T (2021). The genome sequence of the brown trout, Salmo trutta Linnaeus 1758. Wellcome Open Res..

[34] Christensen KA (2018). Chinook salmon (Oncorhynchus tshawytscha) genome and transcriptome. PLoS One..

[35] Gao G (2023). The generation of the first chromosome-level de novo genome assembly and the development and validation of a 50K SNP array for the St. John River aquaculture strain of North American Atlantic salmon. G3 (Bethesda)..

[36] Gertz EM (2006). Composition-based statistics and translated nucleotide searches: improving the TBLASTN module of BLAST. BMC biology..

[37] Slater GSC, Birney E (2005). Automated generation of heuristics for biological sequence comparison. BMC bioinformatics..

[38] Grabherr MG (2011). Trinity: reconstructing a full-length transcriptome without a genome from RNA-Seq data. Nature biotechnology..

[39] Zhang H (2024). The haplotype-resolved genome assembly of autotetraploid rhubarb Rheum officinale provides insights into its genome evolution and massive accumulation of anthraquinones. Plant Commun..

[40] Chan, P. P. & Lowe, T. M. tRNAscan-SE: searching for tRNA genes in genomic sequences. Gene prediction: methods and protocols (Human Press, 2019). 10.1007/978-1-4939-9173-0_1 PMC6768409 31020551

[41] Nawrocki EP, Kolbe DL, Eddy SR (2009). Infernal 1.0: inference of RNA alignments. Bioinformatics..

[42] Delcher AL, Salzberg SL, Phillippy AM (2003). Using MUMmer to identify similar regions in large sequence sets. Current protocols in bioinformatics..

[43] Pfeil BE (2005). Placing paleopolyploidy in relation to taxon divergence: a phylogenetic analysis in legumes using 39 gene families. Systematic biology..

[44] Schmutz J (2010). Genome sequence of the palaeopolyploid soybean. Nature..

[45] Kim J (2016). Multi-tissue transcriptome profiles for coho salmon (Oncorhynchus kisutch), a species undergoing rediploidization following whole-genome duplication. Marine Genomics..

[46] (2023). NCBI Sequence Read Archive.

[47] (2023). NCBI Sequence Read Archive.

[48] (2023). NCBI Sequence Read Archive.

[49] Huang T (2023). Genbank.

[50] (2023). NCBI Sequence Read Archive.

[51] Huang T, Avolio ML (2024). figshare.

