# Supplementary material for: A chromosome-level genome assembly and evolutionary analysis of Coregonus ussuriensis Berg
Source: Sci Data. 2024 Jul 18;11:792. doi: 10.1038/s41597-024-03642-0 (PMC11258136; doi:10.1038/s41597-024-03642-0)
Supplement: Supplementary file 1 — Tables [file 41597_2024_3642_MOESM1_ESM.pdf]

Figure S1 The plot of chromosome-by-chromosome inter-genomic comparison of *Coregonus ussuriensis* vs *Coregonus* sp. *Balchen*.

Figure S2 The plot of chromosome-by-chromosome inter-genomic comparison of *Coregonus ussuriensis* vs *Oncorhynchus mykiss.*

Figure S3 The plot of chromosome-by-chromosome inter-genomic comparison of *Coregonus ussuriensis* vs *Salmo salar.*

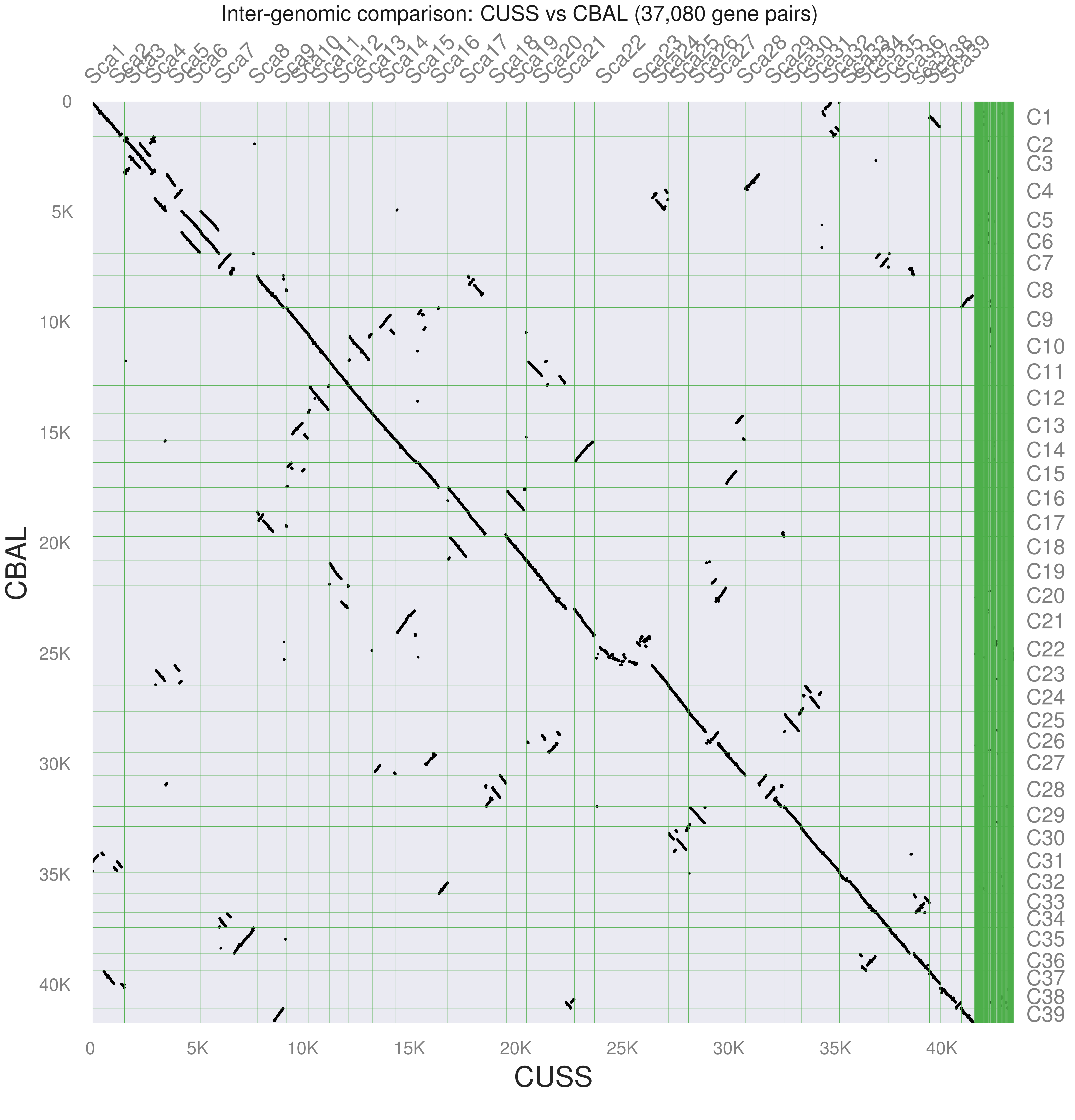

Inter-genomic comparison: CUSS vs CBAL (37,080 gene pairs)
Sca1 Sca2 Sca3 Sca4 Sca5 Sca6 Sca7 Sca8 Sca9 Sca10 Sca11 Sca12 Sca13 Sca14 Sca15 Sca16 Sca17 Sca18 Sca19 Sca20 Sca21 Sca22 Sca23 Sca24 Sca25 Sca26 Sca27 Sca28 Sca29 Sca30 Sca31 Sca32 Sca33 Sca34 Sca35 Sca36 Sca37 Sca38 Sca39
C1 C2 C3 C4 C5 C6 C7 C8 C9 C10 C11 C12 C13 C14 C15 C16 C17 C18 C19 C20 C21 C22 C23 C24 C25 C26 C27 C28 C29 C30 C31 C32 C33 C34 C35 C36 C37 C38 C39
CBAL
0
5K
10K
15K
20K
25K
30K
35K
40K
CUSS
0
5K
10K
15K
20K
25K
30K
35K
40K

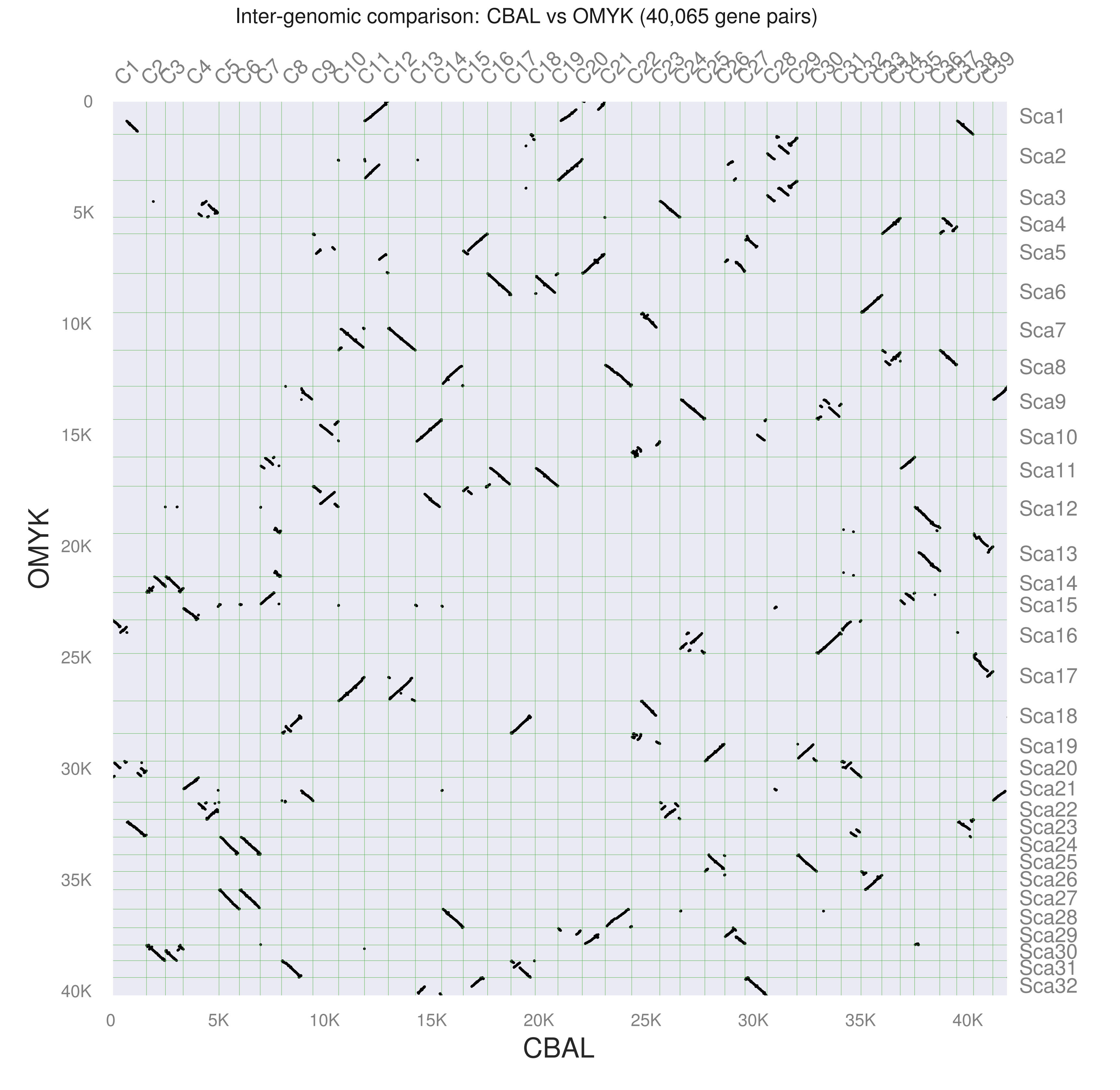

Inter-genomic comparison: CBAL vs OMYK (40,065 gene pairs)
C1 C2 C3 C4 C5 C6 C7 C8 C9 C10 C11 C12 C13 C14 C15 C16 C17 C18 C19 C20 C21 C22 C23 C24 C25 C26 C27 C28 C29 C30 C31 C32 C33 C34 C35 C36 C37 C38 C39
Sca1 Sca2 Sca3 Sca4 Sca5 Sca6 Sca7 Sca8 Sca9 Sca10 Sca11 Sca12 Sca13 Sca14 Sca15 Sca16 Sca17 Sca18 Sca19 Sca20 Sca21 Sca22 Sca23 Sca24 Sca25 Sca26 Sca27 Sca28 Sca29 Sca30 Sca31 Sca32
0
5K
10K
15K
20K
25K
30K
35K
40K
OMYK
CBAL

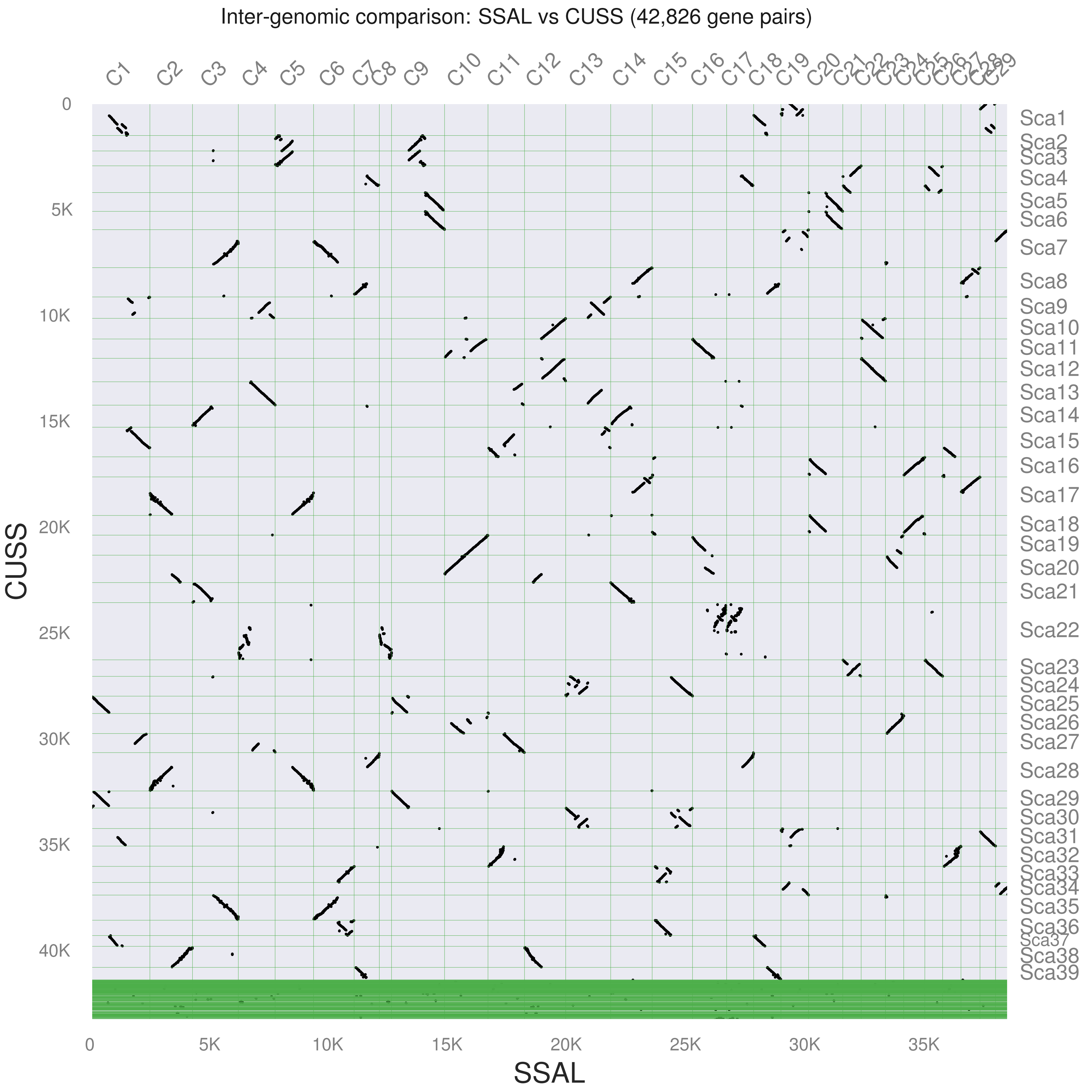
Inter-genomic comparison: SSAL vs CUSS (42,826 gene pairs)
C1
C2
C3
C4
C5
C6
C7
C8
C9
C10
C11
C12
C13
C14
C15
C16
C17
C18
C19
C20
C21
C22
C23
C24
C25
C26
C27
C28
C29
0
5K
10K
15K
20K
25K
30K
35K
40K
CUSS
Sca1
Sca2
Sca3
Sca4
Sca5
Sca6
Sca7
Sca8
Sca9
Sca10
Sca11
Sca12
Sca13
Sca14
Sca15
Sca16
Sca17
Sca18
Sca19
Sca20
Sca21
Sca22
Sca23
Sca24
Sca25
Sca26
Sca27
Sca28
Sca29
Sca30
Sca31
Sca32
Sca33
Sca34
Sca35
Sca36
Sca37
Sca38
Sca39
0
5K
10K
15K
20K
25K
30K
35K
SSAL
